# Carbon- versus sulphur-based zinc binding groups for carbonic anhydrase inhibitors?

**DOI:** 10.1080/14756366.2018.1428572

**Published:** 2018-02-02

**Authors:** Claudiu T. Supuran

**Affiliations:** Neurofarba Department, Sezione di Chimica Farmaceutica e Nutraceutica, Università degli Studi di Firenze, Florence, Italy

**Keywords:** Carbonic anhydrase, inhibitors, inhibition mechanism, zinc binder, anchoring to zinc-coordinated water, sulphonamide, hydroxamate, carboxylate

## Abstract

A set of compounds incorporating carbon-based zinc-binding groups (ZBGs), of the type PhX (X = COOH, CONH_2_, CONHNH_2_, CONHOH, CONHOMe), and the corresponding derivatives with sulphur(VI)-based ZBGs (X = SO_3_H, SO_2_NH_2_, SO_2_NHNH_2_, SO_2_NHOH, SO_2_NHOMe) were tested as inhibitors of all mammalian isoforms of carbonic anhydrase (CA, EC 4.2.1.1), CA I–XV. Three factors connected with the ZBG influenced the efficacy as CA inhibitor (CAI) of the investigated compounds: (i) the pKa of the ZBG; (ii) its geometry (tetrahedral, i.e. sulphur-based, versus trigonal, i.e. carbon-based ZBGs), and (iii) orientation of the organic scaffold induced by the nature of the ZBG. Benzenesulphonamide was the best inhibitor of all isoforms, but other ZBGs led to interesting inhibition profiles, although with an efficacy generally reduced when compared to the sulphonamide. The nature of the ZBG also influenced the CA inhibition mechanism. Most of these derivatives were zinc binders, but some of them (sulfonates, carboxylates) may interact with the enzyme by anchoring to the zinc-coordinated water molecule or by other inhibition mechanisms (occlusion of the active site entrance, out of the active site binding, etc.). Exploring structurally diverse ZBGs may lead to interesting new developments in the field of CAIs.

## 1. Introduction

Simple molecules/ions such as CO_2_, bicarbonate and protons are essential in many important physiologic processes in all life kingdoms (*Bacteria*, *Archaea*, and *Eukarya*) and for this reason, relatively high amounts of carbonic anhydrases (CAs, EC 4.2.1.1), the enzymes which use these molecules/ions as substrates, are present in most of the investigated organisms, all over the phylogenetic tree^1–13^. There are seven genetically distinct CA families known to date^4–7^. The α-CAs are present in vertebrates, arthropods, sponges, corals, fungi, protozoa, algae, and cytoplasm of green plants but also in many *Bacteria* species^1^^,^^7–13^. β-CAs are predominantly found in *Bacteria*, algae, and chloroplasts of both mono- as well as dicotyledons, but also in many fungi and some *Archaea*^1–9^. The γ-CAs were found in *Archaea*, *Bacteria*, and plants^1–13^, whereas the δ-, ζ-, and θ-CAs seem to be present only in marine diatoms^2^. The η-CAs were found to date only in protozoa of the *Plasmodium* type^5^. In all these organisms, CAs catalyse the reversible hydration of carbon dioxide to bicarbonate and protons (hydronium ions), and are involved in crucial physiological processes connected with respiration and transport of CO_2_/bicarbonate, pH and CO_2_ homeostasis, electrolyte secretion in a variety of tissues/organs, biosynthetic reactions (e.g. gluconeogenesis, lipogenesis and ureagenesis), bone resorption, calcification, tumourigenicity, and many other physiologic or pathologic processes (thoroughly studied in vertebrates)^1–13^. In algae, plants and some bacteria they play an important role in photosynthesis and other biosynthetic reactions^10^. In diatoms, CAs play a crucial role in carbon dioxide fixation^4^^,^^10^. Many such enzymes from vertebrates, protozoa, fungi, and bacteria are well-known drug targets^1–13^, and their inhibitors possess pharmacologic applications, being clinically used for the management of glaucoma^1^^,^^14^, edema^1^^,^^15^, obesity^16^, epilepsy^17^, hypoxic tumors^1^^,^^2^^,^^18^^,^^19^, idiopathic intracranial hypertension^20^, etc. Ultimately, some human (h) CA isoforms have also been validated as drug targets for cerebral ischemia^21^, neuropathic pain^22^, and arthritis^23^, whereas many such enzymes present in pathogenic organisms may lead to the development of anti-infectives with a new mechanism of action^24^^,^^25^.

**Figure 1.**
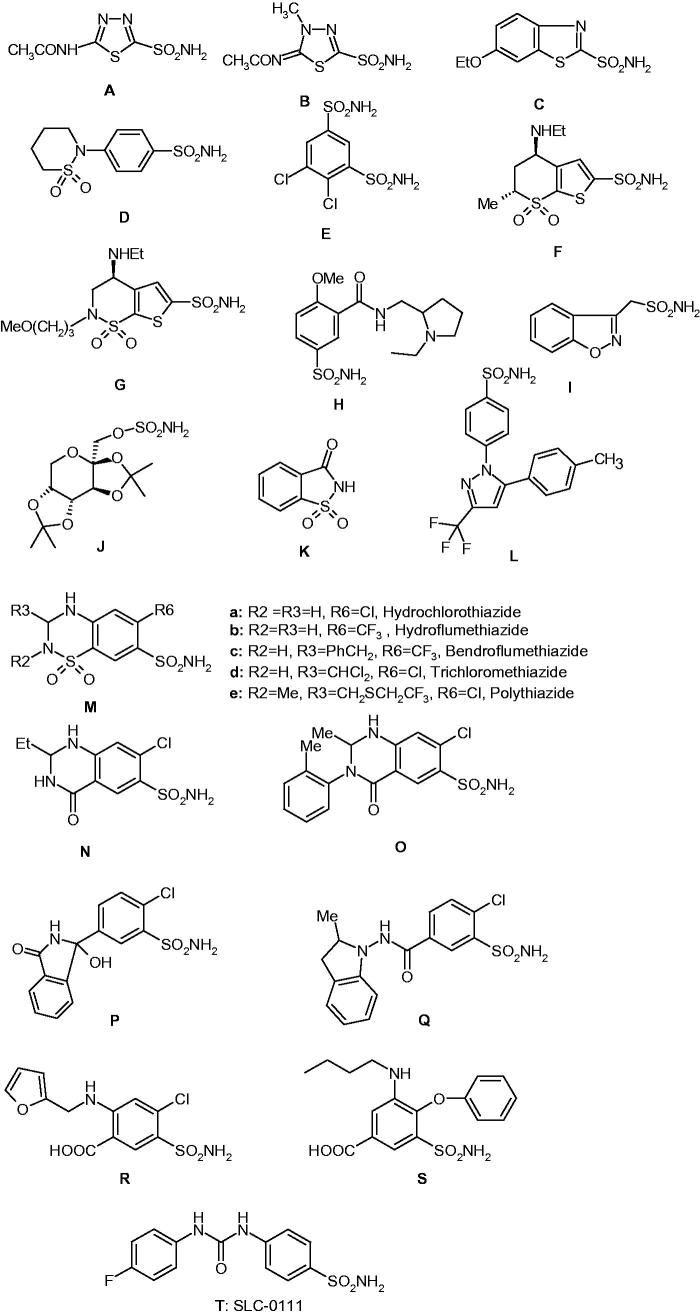
Clinically used sulphonamides/sulphamates with CA inhibitory action (**A–S)** and SLC-0111 (**T**) in phase II clinical trials as an antitumour/antimetastatic agent^18^^,^^19^.

The main class of CA inhibitors (CAIs) is constituted by the primary sulphonamides and their isosteres (sulphamates, sulphamides)^1–6^. They bind in deprotonated form, as sulphonamidate anions, to the metal ion from the enzyme active site, which is most frequently but not in all CA classes, a zinc ion^1–6^^,^^12^^,^^13^. Sulphonamides are the most important class of CAIs^1^^,^^2^^,^^5^^,^^6^^,^^8^^,^^16–19^, since at least 20 such compounds are in clinical use for decades whereas one of them is in clinical development in the last period – Figure 1^1–6^^,14–25^. They include acetazolamide **A**, methazolamide **B**, ethoxzolamide **C**, sulthiame **D**, dichlorophenamide **E**, dorzolamide **F**, brinzolamide **G**, sulpiride **H,** zonisamide **I**, topiramate **J** (a sulphamate), saccharin **K**, celecoxib **L**, chlorothiazide **M** and structurally related highceiling diuretics of types **N, O, P, Q, R,** and **S**, including the widely prescribed hydrochlorothiazide **Ma**, furosemide **R**, bumethanide **S**, all of them being compounds in clinical use for many years, as diuretics, antiglaucoma agents, or antiepileptics^1–6^^,^^14^^,^^26–28^. Compound **T,** SLC-0111, is in phase II clinical trials as an antitumor/antimetastatic agent, and was discovered in the author's laboratory^18^^,^^19^^,^^27^ (Figure 1). Most of sulphonamides **A–S** act as potent CAIs and are in clinical use for decades, but their main problem is related to the fact that by inhibiting most of the catalytically active CA isoforms found in vertebrates (13 such isoforms are known to date^1–13^), they show a wide range of side effects^1–6^^,^^14–19^. Newer generation inhibitors, among which **T** is a good example, were structurally-based drug designed in such a way as to act as isoform-selective inhibitors for the transmembrane, tumour-associated isoforms hCA IX and XII (h = human), and as thus, show less side effects compared to the classical CAIs of types **A–S** discussed above^27^. X-ray crystallography of enzyme–inhibitor adducts was exhaustively been used for designing isoform-selective CAIs belonging to the sulphonamide, sulphamate, and sulphamide classes^12^^,^^13^^,^^26–28^.

**Figure 2.**
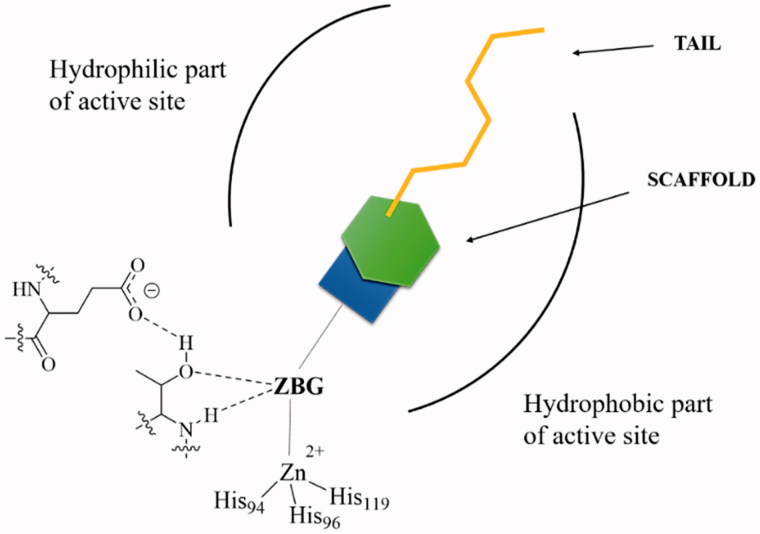
General scheme showing CAIs belonging to the zinc binders class (sulphonamides, sulphamates, sulphamides, carboxylates, hydroxamates, phosphonates, borols, etc.) in interaction with an α-CA^1^^,^^3^. The ZBG is coordinated to the metal ion and makes hydrogen bonds with the gate keeper residues Thr199–Glu106, conserved in all α-CAs^1–3^. The scaffold of the inhibitor may occupy either the hydrophylic or hydrophobic (or both) halves of the active site^12^, whereas the tails are orientated towards the exit of the cavity where the most variable amino acid residues among the different mammalian CAs are located^26–28^. The interactions between the scaffold/tail with the enzyme are not shown.

However, in the last period, many other compounds with a similar mechanism of action were reported, among which dithiocarbamates^29^, xanthates^30^, monothiocarbamates^31^, some carboxylates^32^, hydroxamates^33^, phosphonates^34^, and borols^35^. All these CAIs are collectively termed as zinc binders, and their inhibition mechanism towards α-CAs is shown in Figure 2. These CAIs bind in deprotonated form, as anions, to the Zn(II) ion from the enzyme active site, which is in a tetrahedral geometry, being coordinated by three His residues from the enzyme and by the zinc-binding group (ZBG) of the inhibitor (Figure 2). There is overwhelming X-ray crystallographic evidence showing this type of binding for sulphonamides, sulphamates, sulphamides, dithiocarbamates, and their derivatives, hydroxamates, some carboxylates, one phosphonate and some borols^12^^,^^13^^,^^26–35^. The ZBG also interacts with two other conserved residues in all α-CAs, acting as “gate keepers”, i.e. Thr199 (hydrogen bonded through its OH group with the water molecule/hydroxide ion coordinated to the zinc in the uninhibited enzyme, and with the ZBG, as shown in Figure 2, in the enzyme–inhibitor adducts) and Glu106, which is hydrogen bonded to Thr199 through its carboxylate moiety (Figure 2)^21^^,^^44–46^,57–65. In other CA genetic families, the gate keepers are diverse amino acid residues, but the mechanism described above for α-class enzymes is probably valid (with small variations) for the remaining CA families^1–7^^,^^12^.

In addition to the ZBG, which is highly important in the design of CAIs, an inhibitor, as shown in Figure 2, also possesses a scaffold and a tail. The scaffold is generally an aromatic, heterocyclic, aliphatic, sugar, or a combination of these moieties^1–3^. The tail started to be incorporated in the structure of CAIs starting with 1999, with the discovery of the tail approach^26–28^, and has the role to assure isoform selectivity to the inhibitors, since these moieties, of a highly variable structural type, interact with the residues at the entrance of the active site cavity, which are the most variable regions among the various CA isoforms found in mammals^1–3^^,^^12^^,^^26–28^. Up until now, with few exceptions mentioned above (dithiocarbamates^27–31^, carboxylates^32^, hydroxamates^33^, phosphonates^34^, and borols^35^), the most successful strategies for obtaining potent and selective CAIs were based on ZBGs incorporating sulphur(VI) derivatives, basically variations on the sulphonamide theme^1–3^^,^^12^^,^^13^^,^^26–28^. In this article, I will present a comparison of sulphur-based versus carbon-based ZBGs for designing CAIs, and how the nature of this structural fragment from the inhibitor molecule is in fact the main factor influencing the inhibition profile, not only due to the coordination to the metal ion, but also due to structural constraints which the scaffolds experience when the ZBG is attached to the inhibitor molecule.

## 2. Materials and methods

### 2.1. Chemistry

Compounds **1–10** investigated here as CAIs were prepared as reported in the literature^36–38^, by reaction of phenylsulphonyl chloride or benzoyl chloride (from Sigma-Aldrich, Milan, Italy) with nucleophiles (water, ammonia, hydrazine hydrate, hydroxylamine hydrochloride, O-methyl-hydroxylamine, all commercially available from Sigma-Aldrich, Milan, Italy). These compounds are known in the literature and their physico-chemical and spectral data confirmed their structures (data not shown)^36–38^.

### 2.2. Carbonic anhydrase inhibition assay

An Applied Photophysics stopped-flow instrument has been used for assaying the CA catalysed CO_2_ hydration activity^39^. Phenol red (at a concentration of 0.2 mM) has been used as indicator, working at the absorbance maximum of 557 nm, with 20 mM Hepes (pH 7.5) as buffer, and 20 mM Na_2_SO_4_ (for maintaining constant the ionic strength), following the initial rates of the CA-catalysed CO_2_ hydration reaction for a period of 10–100 s. The CO_2_ concentrations ranged from 1.7 to 17 mM for the determination of the kinetic parameters and inhibition constants. For each inhibitor, at least six traces of the initial 5–10% of the reaction have been used for determining the initial velocity. The uncatalysed rates were determined in the same manner and subtracted from the total observed rates. Stock solutions of inhibitor (10 mM) were prepared in distilled-deionised water and dilutions up to 0.1 nM were done thereafter with the assay buffer. Inhibitor and enzyme solutions were preincubated together for 15 min at room temperature prior to assay, in order to allow for the formation of the enzyme–inhibitor (E–I) complex. The inhibition constants were obtained by non-linear least-squares methods using PRISM 3 and the Cheng–Prusoff equation, as reported earlier^17^^,^^22–28^ and represent the mean from at least three different determinations. All CA isoforms were recombinant ones obtained in-house as reported earlier^22–28^.

## 3. Results and discussion

In order to investigate how the ZBGs based on two different elements, i.e. sulphur and carbon, influence the potency as CAI of the compounds incorporating them, five sulphur(VI) derivatives based on the benzenesulphonic acid **1** and the same number of carbon(IV) derivatives, based on benzoic acid **6**, were investigated as inhibitors of all catalytically active mammalian isoforms, CA I–XV. For both series, the amide (compounds **2** and **7**, respectively), hydrazide (**3** and **8**), hydroxylamide (**4** and **9**) and *O*-methoxylated amide (**5** and **10**) were considered as possible CAIs, and were investigated for their interaction with the human (h) hCA I-XI, and hCA XIV, as well as the murine mCA XIII and mCA XV (Table 1).

**Table 1.** Inhibition of CA isozymes I–XV (of human = h, and murine = m origin) with compounds **1**–**10**.


		*K*_I_ (μM)^a^^,^^b^												
No.	X	hCA I	hCA II	hCA III	hCA IV	hCA VA	hCA VB	hCA VI	hCA VII	hCA IX^c^	hCA XII^c^	mCA XIII	hCA XIV	mCA XV
**1**	OH	8.65	9.30	6.63	9.10	80.9	79.8	8.80	7.93	9.55	6.45	8.72	8.79	8.03
**2**	NH_2_	0.086	0.101	2.26	7.96	1.68	8.82	0.097	0.095	0.097	0.090	0.100	0.092	0.100
**3**	NHNH_2_	9.8	5.31	6.28	78.4	7.75	95.2	74.4	8.56	51.7	2.42	5.96	3.81	5.16
**4**	NHOH	2.73	5.47	4.42	24.6	5.28	67.9	86.2	8.73	60.3	1.53	4.97	1.66	7.17
**5**	NHOMe	>1000	8.96	5.72	39.5	6.70	10.7	27.0	9.56	64.3	8.32	6.21	1.57	7.84
**6**	OH	730	30.1	35.7	434	7.12	9.60	91.6	92.2	66.5	20.4	44.8	0.98	72.8
**7**	NH_2_	324	93.2	35.7	381	46.8	83.1	82.6	76.8	3.67	11.2	45.1	27.0	85.5
**8**	NHNH_2_	91.2	231	40.2	335	59.3	75.9	63.5	76.7	48.4	45.0	44.4	0.99	94.2
**9**	NHOH	83.1	179	44.8	84.7	67.4	53.6	78.1	70.7	45.9	9.51	23.0	0.94	79.9
**10**	NHOMe	72.5	341	37.3	111	89.1	57.3	89.9	72.2	57.5	28.5	63.3	10.0	106

^a^Errors in the range of ±5% of the reported data from three different assays.

^b^h = human; m = murine isozyme.

^c^Catalytic domain.

It may be observed that the scaffold of all these compounds **1–10** was identical, i.e. the simple phenyl moiety, whereas the ZBGs incorporate the central element in tetrahedral geometry for the sulphur derivatives **1–5**, and in trigonal, planar geometry for the carbon ones **6–10** (as the central carbon atom has the sp^2^ hybridization). Thus, both the geometry and the acid-base properties of the ZBGs differ considerably among these compounds, due to these diverse hybridizations and electronic properties of the central element. Indeed, sulphonic acids such as **1** are very strong acids, with pKa in the negative range (**1** has a pKa of –2.8), whereas benzoic acid has a pKa of 4.2^40^. The same differences exist for their derivatives, with the aromatic sulphonamides behaving as much stronger acids (pKa of **2** is of 10.1^41^) than the corresponding amides (pKa of **6** is of around 14.5^40^). Thus, the main differences between the sulphur-based versus the carbon based ZBGs are: (i) geometry of the ZBG due to the diverse hybridization of the main element found in it; (ii) pKa of the ZBG. Both these two factors are known to be relevant for compounds to efficiently bind to Zn(II) in CAs and act as CAIs^1–13^.

The following structure–activity relationship (SAR) was observed from the data of Table 1:

- hCA I was efficiently inhibited by the primary sulphonamide **2**, with an inhibition constant of 86 nM, whereas the remaining derivatives showed a much weaker inhibitory action. For the sulphur-based ZBGs, the *N*-hydroxy sulphonamide **4** and the sulphonic acid **1** were the next best inhibitors after **2**, with *K*_I_s of 2.73–8.65 µM, whereas the *N*-amino and *N*-methoxy sulphonamides **3** and **5** were much less efficient CAIs compared to **2** (*K*_I_s of 69.8 to >1000 µM). Benzoic acid **6** was a very weak inhibitor (*K*_I_ of 730 µM) but all its derivatives **7–10** showed an enhanced activity (*K*_I_s of 72.5–324 µM). In fact, the X-ray crystal structure of the hydroxamic acid **9** bound to hCA II^38^ (the adduct with isoform hCA I was not yet characterised by means of crystallography) showed that the hydroxamate ZBG is bidentate, unlike the sulphonamide or carboxylate ones, with both oxygens present in the CONHOH fragment being coordinated to the catalytic zinc ion, confirming thus the possibility to use this ZBG for designing efficient CAIs.
- hCA II was also best inhibited by the primary sulphonamide **2**, with an inhibition constant of 101 nM, whereas all the remaining sulphur-based derivatives showed micromolar inhibition, with *K*_I_s in the range of 5.31–9.30 µM. For this isoform, there are not relevant differences of activity between the NHOH and NHNH_2_ substituted derivatives **3** and **4**, which showed very similar inhibitory action. The *N*-methoxysulphonamide **5** was on the other hand very similar in its inhibitory behaviour to the sulphonic acid **1** (*K*_I_s of 8.96–9.30 µM). In fact, the X-ray crystal structures of **2, 3,** and **4** bound to this isoform are available^37^^,^^38^^,^^42^, showing that although the interaction between the ZBG and the metal ion and amino acid residues in its neighbourhood are different for the three compounds, all of them lead to efficient inhibitors due to various factors which will be discussed in detail shortly. For the carbon-based ZBGs, the best inhibition was observed for the carboxylate **6** (*K*_I_ of 30.1 µM), followed by the carboxamide **7** (*K*_I_ of 93.2 µM), whereas the remaining derivatives were much weaker CAIs (*K*_I_s of 179–341 µM, Table 1). Thus, for hCA II (as for hCA I), there is a net loss of CAI activity in the carbon-based ZBGs compared to the corresponding sulphur-based ones.
- hCA III was inhibited in the low micromolar range by the sulphur-containing compounds **1–5** (*K*_I_s of 2.26–6.63 µM) and in the high micromolar range by the carbon-based derivatives **6–10** (*K*_I_s of 35.7–44.8 µM). It is interesting to note the rather limited range of variation for the *K*_I_s for both subseries, meaning that the nature of the ZBG does not influence inhibitory potential so strongly for this isoform compared to hCA I and II discussed above. Indeed, the sulphonic acid **1** and the sulphonamide **2** only differ by a factor of 3 in their inhibition against hCA III (whereas for hCA I the sulphonamide **2** was 100 times a better inhibitor compared to the sulphonic acid **1**, see Table 1). Even more striking were the results for the carbon based ZBG, with the carboxylic acid **6** and the carboxamide **7** having practically the same inhibition constant.
- hCA IV, a membrane-bound isoform, was also better inhibited by the sulphur-based than the carbon-based derivatives. For the first subseries, again the sulphonamide and the sulphonic acid were the most efficient inhibitors, with *K*_I_s of 7.96–9.10 µM, whereas the *N*-amino, *N*-hydroxy-, and *N*-methoxy sulphonamides were less effective, with *K*_I_s of 24.6–78.4 µM. For the second subseries, the hydroxamate **9** was the best inhibitor (*K*_I_ of 84.7 µM) whereas the remaining derivatives were much less effective (*K*_I_s of 111–434 µM). As for hCA I and II, there are net differences of activity between the sulphur-based and the carbon-based inhibitors.
- For the first mitochondrial isoform, hCA VA, the most efficient inhibitors were the benzenesulphonamide **2** and its *N*-substituted derivatives **3–5**, with *K*_I_s of 1.68–7.75 µM, whereas the sulphonic acid **1** was a much weaker inhibitor (*K*_I_ of 80.9 µM). For the carbon-based compounds, the carboxylate **6** was a micromolar inhibitor (*K*_I_ of 7.12 µM), thus almost as active as some of the sulphur-containing compounds (derivatives **2–5**). On the other hand, derivatives **7–10** were around one order of magnitude less efficient hCA VA inhibitors compared to **6**, with *K*_I_s of 46.8–89.1 µM.
- The second mitochondrial isoform hCA VB had a different inhibition profile compared to hCA VA, with which it has a rather high homology^1–4^. Thus, the primary sulphonamide **2**, its *N*-methoxy-derivative **5** and benzoic acid **6** showed the best inhibitory action, with *K*_I_s of 8.82–10.7 µM, whereas all the remaining derivatives were less efficient inhibitors, with *K*_I_s ranging between 53.6 and 95.2 µM. In this case, a similar behaviour of some of the sulphur-based and carbon-based ZBG-containing compounds was observed, such as, for example, the SO_2_NHNH_2_ and CONHNH_2_ pairs (**3** and **8**), or SO_2_NHOH and CONHOH derivatives **4** and **9**, which possess similar inhibitory action (Table 1).
- The secreted isoform hCA VI was potently inhibited by sulphonamide **2** (*K*_I_ of 97 nM), with the next most efficient inhibitor being the sulphonic acid **1** (*K*_I_ of 8.80 µM). All other compounds were much weaker inhibitors, with *K*_I_s in the range of 27.0–91.6 µM (Table 1).
- The brain-associated cytosolic isoform hCA VII was also efficiently inhibited by the sulphonamide **2** (*K*_I_ of 95 nM), whereas the remaining compounds with sulphur-based ZBGs showed a very similar behaviour of low micromolar inhibitors (*K*_I_s of 7.93–9.56 µM). All the carbon-based compounds were on the other hand high micromolar inhibitors (*K*_I_s of 70.7–92.2 µM).
- The tumour-associated transmembrane isoform hCA IX was efficiently inhibited by sulphonamide **2** (*K*_I_ of 97 nM), and surprisingly, the next most efficient inhibitor was the carboxamide **7**, with a *K*_I_ of 3.67 µM. Apart the sulphonic acid **1**, which was a low micromolar inhibitor, the remaining derivatives showed inhibition constants in the high micromolar range, of 45.9–66.5 µM (Table 1). Many of the carbon-based ZBGs were thus more efficient in producing tighter binders for hCA IX compared to the sulphur-based ZBGs, which is a quite surprising result.
- The second transmembrane, tumour-associated enzyme hCA XII was again best inhibited by the sulphonamide **2** (*K*_I_ of 90 nM), whereas all the remaining sulphur-containing compounds were medium potency inhibitors, with inhibition constants in the range of 1.53–8.32 µM. Among the carbon-based derivatives, the best inhibitor was the hydroxamate **9** (*K*_I_ of 9.51 µM) and the amide **7** (*K*_I_ of 11.2 µM), with the remaining compounds acting as less efficient inhibitors (inhibition constants in the range of 20.4–45.0 µM).
- mCA XIII, a cytosolic isoform quite similar to the corresponding human enzyme^1–4^, was best inhibited by the sulphonamide **2** (*K*_I_ of 100 nM), with the remaining sulphur derivatives **1, 3–5** acting as low micromolar inhibitors (*K*_I_s of 4.97–8.72 µM). The carbon-based inhibitors **6–10** were less effective for inhibiting mCA XIII, with *K*_I_s in the range of 23.0–63.3 µM.
- hCA XIV, another transmembrane isoform, was also efficiently inhibited by the primary sulphonamide **2** (*K*_I_ of 92 nM), but many of the carbon-based compounds also showed submicromolar inhibitory action. Indeed, benzoic acid **6**, its hydrazide **8** and hydroxamate **9**, showed *K*_I_s in the range of 0.94–0.99 µM. However, the amide **7** and the methylated hydroxamate **10** were weaker inhibitors, with inhibition constants in the range of 10.0–27.0 µM. The compounds with sulphur-containing ZBGs (except the sulphonamide discussed above) were low micromolar inhibitors, with *K*_I_s in the range of 1.57–8.79 µM.
- mCA XV (the isoform XV is not present in primates, where it is encoded by a pseudogene, but is present in other vertebrates, including rodents^1–4^) was efficiently inhibited by the sulphonamide **2** (*K*_I_ of 100 nM), whereas the remaining sulphur-based derivatives were micromolar inhibitors (*K*_I_s in the range of 5.16–8.03 µM). The carbon-based compounds were much less efficient inhibitors, with *K*_I_s in the range of 72.8–106 µM (Table 1).
- The inhibition profiles of the investigated mammalian isoforms with both types of inhibitors were very different, with the same compound showing a range of inhibitory potencies. For example, although the primary sulphonamide was the best inhibitor of all isoforms, its efficacy ranged between 86 nM (for the most sensitive isoform, hCA I) and 8.82 µM (for the least inhibited isoform, hCA VB), i.e. differing by a factor of >100. This situation was in fact observed for most other compounds investigated here, showing thus that a series of different factors, not only pKa and geometry of the ZBG may influence the binding efficacy of the compound to the active site.

## 4. Conclusions

**Figure 3.**
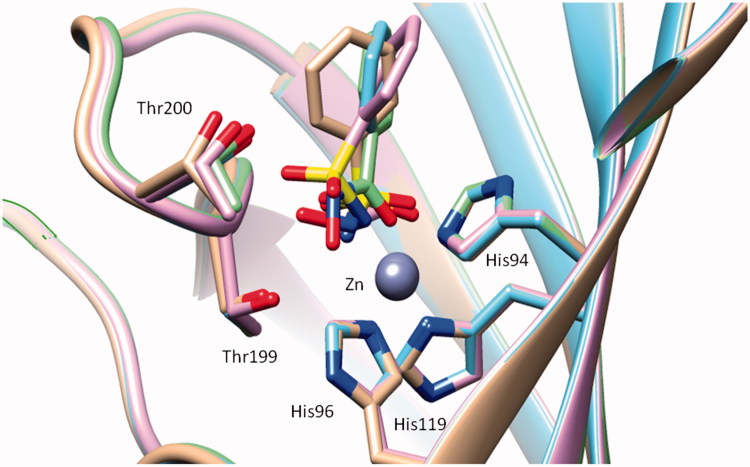
Superimposition of the hCA II – **2** adduct (blue, PDB code 2WEJ^42^) with the hCA II – **4** adduct (violet, PDB code 3T5U^37^), the hCA II – **5** adduct (silver, PDB code 3T5Z^37^) and the hCA II – hydroxamate **9** adduct (green, PDB code 4FL7^38^).

By analysing the inhibitory action of the 10 structurally related derivatives towards the 13 diverse mammalian CA isoforms (Table 1), one can observe that the inhibition efficacy is a multifactorial process, not easy to rationalise, even for these relatively simple compounds for which the scaffold was constant (a phenyl moiety). However, the X-ray crystal structures for the adducts of four of the considered compounds discussed here bound to hCA II, the sulphonamide **2**^42^ (PDB code 2WEJ), the *N*-hydroxysulphonamide **4** (PDB code 3T5U^37^), the *N*-methoxy-sulphonamide **5** (PDB code 3T5Z^37^) and the hydroxamate **9** (PDB code 4FL7^38^) are known and they were crucial for rationalising the results presented here. Considering these data, it appears that at least three factors connected with the ZBG influence the efficacy as CAI of the investigated compounds:

(i) *The pKa of the ZBG*, which as mentioned above, varied on almost 20 pKa units, from the highly acidic sulphonic acid **1** to the poorly acidic amide **7**. The way in which this parameter influences CAI properties was in fact highly debated already from the first phases of research in the CAIs field^1–4^, as it is not so straightforward how this parameter influences activity. Indeed, a zinc binder must be deprotonated for efficiently binding the zinc ion (Figure 1). However, the highly acidic sulphonic acid **1** (pKa of –2.8) is for example a much weaker CAI compared to the poorly acidic sulphonamide **2** (pKa of 10.1). Thus, pKa is not the only parameter influencing CAI effects, or probably it is not the most influential factor. An effective zinc binding inhibitor must dissociate easily to form the anion, but it should not be a too strong or a too weak acid. This is why probably both the sulphonic acids and the amides are weaker CAIs compared to the sulphonamides.

(ii) *The geometry of the ZBG*. The tetrahedral, i.e. sulphur-based, and the trigonal, i.e. carbon-based ZBGs, are able to bind in a diverse manner to the amino acid residues in the neighbourhood of the metal ion, such as Thr199 (and Thr200) and to the metal ion itself^1–4^^,^^37^^,^^38^. Indeed, the sulphur-based ZBGs seem to always bind in a monodentate fashion to the metal ion, probably also due to the fact that the sulphonamide and its *N*-substituted derivatives are more acidic than the corresponding *N*-substituted carbon-derived ZBGs of the carboxamide type (except the COOH, which is of course more acidic than the sulphonamide moiety). For example, the hydroxamate **9**, as mentioned earlier, is one of the few bidentate ZBGs known to date among CAIs, and binds in deprotonated form to the zinc ion by means of both of its oxygen atoms from the CONHO^–^ moiety^38^. On the other hand, both sulphur-based and carbon-based ZBGs also participate in a strong hydrogen bond with Thr199, an amino acid residue conserved in all α-CAs^1–4^. As seen from Figure 3, the four ZBGs present in these inhibitors (except for the bulky SO_2_NHOMe one) are rather superimposable. However, the way in which they interact with Zn(II) and residues at the bottom of the active site is rather diverse, as seen from Figure 3. For the three sulphonamide-based ZBGs, the metal ion is coordinated monodentately by the deprotonated N^–^ atom of the ZBG, and a hydrogen bond with the OH of Thr199 further stabilises the adduct. The hydroxamate ZBG, as mentioned earlier, is also deprotonated but acts as a bidentate ligand (Figure 3).

(iii) *The orientation of the organic scaffold induced by the nature of the ZBG*, is the last factor influencing activity, and is probably the most unexpected one. This is obvious from the superimposition shown in Figure 3. It may be seen that the nature of the ZBG present in these four inhibitors leads to a rather diverse orientation of the phenyl scaffold in some of them. Thus, the phenyl scaffolds of the four inhibitors shown in Figure 3 are not at all superimposable, and this is mainly due to the influence that the ZBG has on the scaffold. It may be seen that a dramatic tilting of the phenyl group in **5** and **9** (compared to **2** and **4**) leads to different orientations of the scaffold in these CAIs. But can we exploit these facts for designing other types of CAIs? I will present some examples which demonstrate that these findings can indeed lead to new developments in the field. In fact, the presence of a heteroatom (or a small functionality) between the organic scaffold and the ZBG may induce a diverse orientation of the scaffold, with profound consequences for the activity of the CAI. This is well illustrated for the examples given below in Table 2. Introduction of an oxygen atom or a NH moiety between the ZBG and the phenyl ring in sulphonamide **2** leads to the sulphamate **2a** and sulphamide **2b**. These two derivatives were tested for the inhibition of 4 CA isoforms^43a,b^, and as seen from data of Table 2, they are much more potent CAIs compared to the lead **2**. The sulphamate **2a** is a low nanomolar inhibitor of the four CA isoforms, and the sulphamide **2b**, although slightly less effective than the sulphamate is also a very potent CAI. Although there are no X-ray crystal structures of **2a** and **2b** bound to CA II (to be compared with the hCA II – **2** adduct), presumably the extra O and NH moieties present in these compounds lead to a diverse orientation of the scaffold when the compounds are bound to the enzyme. This was in fact well documented for the sulphamide analogue of topiramate **J** (possessing the NHSO_2_NH_2_ instead of the OSO_2_NH_2_ ZBG)^43c^ which is a much weaker CAI compared to **J**, and its X-ray crystal structure in adduct with hCA II showed a completely different binding mode^43c^ compared to topiramate in its hCA II adduct^43d^.

**Table 2.** hCA I, II, IX, and XII inhibition data with compounds **2, 2a, 2b, 7,** and **7a**.


	*K*_I_ (µM)^c^			
Compound	hCA I	hCA II	hCA IX	hCA XII
**2**	0.086	0.101	0.097	0.090
**2a**	0.002^a^	0.0013^a^	0.063^a^	0.008
**2b**	0.013^b^	0.012^b^	0.071	0.014
**7**	324	93.2	3.67	11.2
**7a**	720	110	10.1	2.81

^a^From ref.^43a^

^b^From ref.^43b^

^c^Errors in the range of ±5% of the reported data from three different assays.

On the other hand, introduction of the oxygen atom between the scaffold and the ZBG in amide **7** led to the carbamate **7a**, which was also tested as CAI against four CA isoforms (Table 2). As seen from data of Table 2, in this case only hCA XII was better inhibited by the carbamate **7a** compared to the carboxamide **7**, whereas for the remaining isoforms the carboxamide was a better inhibitor compared to the carbamate. However, both derivatives act as rather weak CAIs compared to the sulphonamide, sulphamate, and sulphamide **2, 2a,** and **2b** (Table 2), but probably their activity may be optimised by choosing scaffolds which interact in a favourable manner with the enzyme active site.

Another aspect, which has not been contemplated in detail here due to lack of X-ray crystallographic data, is the fact that the nature of the ZBG may even induce a diverse inhibition mechanism. In fact, the zinc binders are just one and the best studied example of CAIs, but work in the last 10 years led to the discovery of several non-classical inhibition mechanisms, such as the anchoring of the inhibitor to the zinc-coordinated water molecule/hydroxide ion^44–46^, occlusion of the active site cavity entrance^47^ and out of the active site binding^32f^. Not to mention that there are many classes of potent CAIs (e.g. tertiary and secondary sulphonamides are the most investigated ones) for which the inhibition mechanism is not yet elucidated^48^^,^^49^. For example, a sulphonic acid derivative obtained by the CA mediated hydrolysis of a sulphocoumarin, was shown to anchor to the zinc-coordinated water molecule^44^, opening the possibility that the sulphonic acid **1** investigated here may have a similar inhibition mechanism. The carboxylates are probably the most complicated family of CAIs since a variety of inhibition mechanisms were reported for many of them. In fact, some carboxylates coordinate to the metal ion in a monodentate fashion^32^, others were observed anchored to the zinc coordinated water^32^, others were found at the entrance of the active site^47^, and one such derivative even outside the active site cavity^32f^. Thus, it is unknown at this moment whether compound **6** (and also its derivatives **7, 8,** or **10**) inhibits CAs by the classical mechanism shown in Figure 2 or by alternative mechanisms. It is even possible that different isoforms are inhibited by different mechanisms with the same compound, as recently exemplified for acesulfame potassium salt, which is coordinated to the zinc ion in its complex with CA IX and anchored to the water coordinated to the metal ion in the CA II adduct^50^.

The conclusion of all these data is that although the sulphonamide seems to be the best ZBG for generating effective inhibitors of all CA isoforms, other ZBGs investigated here afforded interesting inhibition profiles, with efficacies reduced compared to the primary sulphonamide, but probably prone to be optimised, if an adequate scaffold is attached to them. Furthermore, as discussed above, the nature of the ZBG also influences the CA inhibition mechanism, with most of the derivatives investigated here probably being zinc binders. However, some of them (sulfonates, carboxylates) may interact with the enzyme by anchoring to the zinc-coordinated water molecule or by other inhibition mechanisms (occlusion of the active site entrance, out of the active site binding, etc.) which are less well investigated for the moment. Thus, exploring structurally diverse ZBGs may lead to many other interesting new developments in the design of isoform-selective CAIs, with the present findings attempting to share more light in this field.
